# Relationship Dynamics of Couples Facing Advanced-Stage Parkinson’s Disease: A Dyadic Interpretative Phenomenological Analysis

**DOI:** 10.3389/fpsyg.2021.770334

**Published:** 2022-01-24

**Authors:** Emilie Constant, Elodie Brugallé, Emilie Wawrziczny, Céline Sokolowski, Charlotte Manceau, Bérengère Flinois, Guillaume Baille, Defebvre Luc, Kathy Dujardin, Pascal Antoine

**Affiliations:** ^1^Université de Lille, CNRS, UMR 9193 – SCALab – Sciences Cognitives et Sciences Affectives, Lille, France; ^2^Centre Hospitalier Regional et Universitaire de Lille, Lille, France

**Keywords:** couple dynamic, advanced Parkinson’s disease, interpretative phenomenological analysis, qualitative study, health psychology

## Abstract

**Background/Objective:**

Several studies have examined the impact of Parkinson’s disease (PD) on the quality of couples’ relationships. To date, few studies have explored how couples experience their relationship dynamic by taking into account the disease stage. The objectives of this study were to understand the experience of each partner and to study the mechanisms that underlie their couple organization in the advanced stage of PD.

**Methods:**

Semistructured individual interviews conducted with fifteen patients and their partners were the subject of a dyadic interpretative phenomenological analysis.

**Results:**

Three themes were identified from the analysis: the first, “A Closeness That Separates,” allows the identification of different patterns of interactions that lead to emotional distancing between the partners; the second, “The Adversity Is Not Unbearable, But Going It Alone Would Be,” emphasizes the importance of how the assisting partner provides support; and the third, “Be Prepared for Anything and Facing an Uncertain Future,” reveals the extent and modes of the dyadic regulation of the emotions linked to what the future may hold.

**Conclusion:**

Psychological support is important to help couples express both their feelings and their respective needs in the evolving context of PD.

## Introduction

Over time, couples are at risk of encountering major life problems, such as one of the partners becoming seriously ill. Parkinson’s disease (PD) is the second most common neurodegenerative disease after Alzheimer’s disease (Feigin et al., 2019). PD is a pathology that affects movement, characterized at the motor level by bradykinesia (reduction in the amplitude and speed of movements) associated with rigidity and/or tremor, often in conjunction with postural disorders. PD patients also exhibit non-motor symptoms that could be at the foreground, such as dysautonomia, anosmia, sleep disorders, and pain, as well as cognitive and psychological-behavioral disorders (Armstrong and Okun, 2020). Hence, PD is a heterogeneous disease with respect to its clinical expression and the patient’s disease experience (Fereshtehnejad and Postuma, 2017). In this context, the afflicted and assisting partners face emotional difficulties that can influence their relationship dynamic.

Several studies have investigated the physical and psychological impacts of PD on patients and on their assisting partners (e.g., Birgersson and Edberg, 2004; Hodgson et al., 2004; Mavandadi et al., 2014; Martin, 2016; Buhmann et al., 2017). However, few studies to date have explored the functioning of the couple, and most of them have taken into account several disease stages, which risks confounding distinct situations in terms of the time spent living with the disease or the intensity of the various symptoms. In light of these two considerations, studying the experience of couples while distinguishing each stage of the disease appears to be important. What is needed is a clear understanding of the mechanisms underlying conjugal functioning that can contribute to an adjustment, both positive and negative, regarding the disease. We expect that the identification of such couple mechanisms will allow care to be developed that is tailored to the specific needs of couples. The aim of this study was to further explore the experiences of couples in their daily lives with regard to PD during the advanced stage of the disease.

The advanced stage of PD starts several years after the onset of the first symptoms, when motor and non-motor fluctuations, as well as dyskinesias induced by dopa therapy, manifest. During this stage, motor and non-motor disorders become severe and more difficult to control with treatments (Fasano et al., 2019). A notable psychological impact is characterized by a decrease in self-esteem (Posen et al., 2000), withdrawal (Hudson et al., 2006) and feelings of loss and uncertainty (Caap-Ahlgren and Dehlin, 2002; Hudson et al., 2006). Patients report more frequent anxiety and depressive symptoms (e.g., Bonnet et al., 2013; Defebvre and Vérin, 2020). Worsening motor symptoms decrease patients’ autonomy and render them increasingly dependent on their partner (Vatter et al., 2018). A deterioration in the quality of life of the latter has also been highlighted as the disease progresses (e.g., Martinez-Martin et al., 2008; Den Oudsten et al., 2011; Martinez-Martin and Kurtis, 2012). In addition, stress factors and emotional distress become more pronounced over time (Carter et al., 1998; D’Amelio et al., 2009). At the couple level, the disease increasingly assumes a more central role in the relationship, and the roles evolve with the disease (Birgersson and Edberg, 2004). The worsening of the symptoms adversely impacts the patient in their daily life, which is compensated for by the assisting partner taking on roles that were previously assumed by the afflicted partner. Becoming aware of these changes required to readjust to daily life raises the issue of the place and role of each person in the relationship (Smith and Shaw, 2017). Thus, different dimensions of the couple are impacted by the progression of the disease, such as communication, emotional and physical intimacy, and shared social activities. The loss of autonomy of the afflicted partner limits the opportunities for outings and reduces the activities shared by the couple (Habermann, 2000). The resulting social and couple isolation has a negative impact on the relationship and quality of life of both individuals (Martin, 2016). An increase in discord between partners with the onset and progression of the disease has been reported in several studies (e.g., Carter et al., 1998; Birgersson and Edberg, 2004).

Continuing with activities as a couple and as an individual appears to be important so that both parties can continue to define themselves by something other than their identity as a patient or a caregiver (Bramley and Eatough, 2005; Barken, 2014). In addition, Martin (2016) reported that when couples communicate about difficulties as a result of PD and address them together through mutual support, the closeness between partners is strengthened. The stress caused by the disease can thus lead to emotional closeness between partners when it is experienced as a couple (Fergus and Skerrett, 2015). Buhmann et al. (2017) reported that the frequency of tenderness between partners increases over time. Non-sexual aspects of a relationship become more important for partners who, over time, construct a new vision of their identity as a couple (Mavandadi et al., 2014).

All of these studies suggest the changes that a couple may go through as a result of PD. Further work is needed to better understand possible dyadic developments and the mechanisms that underlie these changes at a given disease stage. The objective of this study was to explore the experience of couples with advanced PD in accordance with previous research in couples with neurodegenerative diseases (e.g., Wawrziczny et al., 2016a,b, 2019). More specifically, we aimed to explore how both partners experience their couple dynamic, and we sought to identify the mechanisms that underlie the changes, both good and bad, that couples undergo in this context. Both partners were interviewed, and we assumed that the lived experience of each and the meaning that both members of the dyad gave to their own and shared experiences were interdependent.

## Materials and Methods

### Participants

Fifteen couples participated in the present study (Table 1). For eleven couples, the patient was a man. The mean age of the patients was 65.93 years old (SD = 7.39), and that of the partners was 65.27 years old (SD = 6.43). The mean duration from the time of diagnosis to the time of the interview was 12.6 years. The relationship duration ranged from 20 to 56 years, with a mean of 42.4 years.

**TABLE 1:** Participant information.

Couple patient/partner^a^	Age of the patient	Disease duration (years)	Age of the partner	Relationship duration (years)
Aline/Adrien	68	19	71	52
Bertrand/Bérengère	79	14	69	51
Claude/Camille	73	12	72	48
David/Domitille	64	21	62	37
Eloi/Élisabeth	57	7	54	38
François/Fabienne	75	8	75	56
Gaëlle/Grégoire	64	17	65	47
Héloïse/Hugo	67	22	71	49
Isaac/Isabelle	75	15	70	48
Juliette/Julien	55	6	67	29
Karim/Kathy	71	9	66	48
Luc/Ludivine	62	13	64	46
Marc/Mélanie	62	9	60	27
Nicolas/Noémie	59	11	54	40
Oscar/Olivia	58	6	59	20

*^a^Pseudonyms.*

### Procedure

The participants were recruited through the Neurological and Movement Disorders Department of the Lille University Medical Center (France). Clinical characteristics were recorded, including an evaluation of the severity of PD by the Hoehn and Yahr stage (Hoehn and Yahr, 1967) and of overall cognition by the Montreal Cognitive score (Nasreddine et al., 2005). Depression, anxiety and apathy were assessed by the Hamilton Rating Scale for Depression (HAM-D, Hamilton, 1960), the Parkinson Anxiety Scale (PAS, Leentjens et al., 2014), and the Lille Apathy Rating Scale (LARS, Sockeel et al., 2006), respectively (Table 2). The inclusion criteria were as follows: (i) couples living together for at least 5 years, with one of the partners having received a PD diagnosis and being at an advanced stage; (ii) both the patient and the assisting partner were able to physically and cognitively communicate with the interviewer; and (iii) age between 40 and 80 years old. The focus of this study was the dyad. The patient’s neuropsychologist, clinical psychologist or referring neurologist informed the patients and their partner in writing that they were eligible to participate in the present study. After couples provided their written consent, an appointment for the interview was then set with the interviewers at the couples’ home.

**TABLE 2:** Means (M), standard deviations (SD), and ranges of clinical variables.

	Patients		
	M	SD	Range
Disease stage^a^	2.5	0.5	(2–3)
Cognitive status^b^	25.93	2.30	(22–30)
Depression score^c^	7.57	6.01	(0–20)
Anxiety score^d^	7.86	6.21	(0–20)
Apathy score^e^	-24.14	7.49	(–34 to –3)

*^a^The Hoehn and Yahr score was evaluated during treatment (Hoehn and Yahr, 1967).*

*^b^The cognitive status was evaluated using the Montreal Cognitive score (Nasreddine et al., 2005).*

*^c^The depression score was evaluated using the Hamilton Rating Scale for Depression (HAM-D, Hamilton, 1960).*

*^d^The anxiety score was evaluated using the Parkinson Anxiety Scale (PAS, Leentjens et al., 2014).*

*^e^The apathy score was evaluated using the Lille Apathy Rating Scale (LARS, Sockeel et al., 2006).*

In the present study, separate interviews were conducted to create an intimate space and to investigate participants’ own experience with PD as well as unsaid things in their relationships. When we presented the study to the couples, we explained the study design to the partners, and they were aware that we would take into account their respective lived experience in the interpretation of the analysis. The interview began with a general question regarding the participant’s experience with PD, followed by themes addressing individual lived experience, the couple’s history and past functioning; the impact of the disease on couple functioning; and their perspectives on the future. The interviews were conducted by clinical psychologists trained in the interpretative phenomenological analysis (IPA), and in the past, they were involved in different studies in this field. The analysis process was supervised by a psychologist and researcher who is experienced in IPA. In addition, the interviewers were unaware of the clinical details of each patient’s disease and, consequently, had no knowledge of the potential results.

The patients’ interviews lasted an average of 86.89 mins (range: 49–139), and the partners’ interviews lasted 83 mins (range: 44–127). The interviews were audio recorded and transcribed.

The study was approved by the national ethics committee (Committee for the Protection of Persons, CPP Est IV, IDRCB 2017-A0261152).

### Data Analysis

In accordance with the research conducted in couples with a neurodegenerative disease (e.g., Wawrziczny et al., 2016a,b, 2019), the aim of this study was to explore the experience of couples confronted with advanced PD and to put into perspective the patient and his or her partner meaning-making regarding their couple functioning. Therefore, the data were analyzed qualitatively using IPA (Smith and Osborn, 2003). This method is well suited for this analysis because it captures the individual’s lived experience and the meaning-making associated with a given phenomenon (Eatough and Smith, 2008). To study the mechanisms through a multiperspective design (Larkin et al., 2019) in the context of couples that underlie the functioning of couples (e.g., Antoine et al., 2013, 2018), it is important to assess the experience of each of the partners, patient and assisting partner. The analyses were related to the divergences and convergences in the discourses of partners to account for the singularity and the complexity of the individual functioning intertwined in the dyadic functioning. We met with the afflicted partner and the assisting partner individually to allow partners to explore their experience as a couple without introducing bias due to the presence of the other partner.

In the data analysis, we put into perspective each partner’s experience regarding their couple life by focusing on the divergences and convergences between each partner in their interview. The analyses were conducted through the different steps recommended in IPA analysis (Nizza et al., 2021). First, the verbatim transcripts were read several times for familiarization with the data. Second, each psychologist annotated and analyzed the interview transcriptions through an idiographic conceptualization. Hence, the psychologists explored the experience of each partner by focusing on the dyadic processes. Then, they put together into perspective the lived experience of both partners, and they highlighted the salient dyadic process of the couple dynamic. The analyses were performed on a couple-by-couple basis, i.e., when a couple was analyzed, the lived experience and the underlying processes that emerged were assessed in conjunction with the previous cases. After each pair of analyses, the psychologists provided a summary to the supervisor. At the end of the couple analyses, the two psychologists and the supervisor attempted to identify salient elements to achieve a synthesis of the couple experience and the associated dyadic processes. Hence, this process allowed the identification of the typologies of the dyadic functioning underlying the couple’s lived experience while also taking into account the couple functioning prior to the disease.

## Results

### At This Stage, the Disease Binds Us Together

At the advanced stage of the disease, the symptoms have progressed, which leads to an increasing degree of dependence. The couple balance is challenged in two ways: dependence is experienced as an alienation from the other partner, and the feeling of being “bound” together leads to tensions that can result in emotional distancing, as expressed by Adrien and Aline.

**Table:** 

I now feel like I’m too important in the relationship… completely out of balance in fact. In addition, it is true that from time to time she wakes up, she says: “you treat me like a little girl.” In addition, it is true that I really do treat her as if she is ill (Adrien)	It annoys me because… it is true he understands me quite well. Sometimes we are running errands and he says to me “let’s go home, I can tell that you are not well.” I say “no, it is going to be okay,” and he says, “you say that, and then later it is not okay, and then we have to hurry home.” He is much more reasonable than me. In addition, I am also clumsier and slower. However, I also like doing as I please. (…) So I say, “Yes, Dad.” Then, he says, “Alright, that’s enough! I am just trying to help.” I say, “That is right.” (Aline)

*The first names of the assisting partners (left) and the afflicted partners (right) are fictitious. I (interviewer).*

Adrien and Aline manage to express their experiences and thus negotiate in real time the balance between the amount of assistance required and the degree of autonomy, which is not the case for other dyads. The advanced stage of PD gives rise to relationship challenges for most couples. In the first part, “A Closeness That Separates,” we describe different forms of interactions between the partners, and then, in the second part, “The Adversity Is Not Unbearable, But Going It Alone Would Be,” we examine the issues related to the protection of the partners and the couple at this stage. Finally, in the third part, “Be Prepared for Anything and Facing an Uncertain Future,” we describe the modes of varying symmetry for the regulation of the partners’ feelings regarding the future (Table 3).

**TABLE 3:** Summary of themes.

Overarching themes	A closeness that separates	The adversity is not unbearable, but going it alone would be	Be prepared for anything and facing an uncertain future
Subthemes	• The pattern “closeness/withdrawal-resignation” • The pattern “intrusion/withdrawal-impotence” • The pattern “closeness/attack-blame” • The pattern “fusion/contagion-invasion”	• Becoming aware of the difference between “support my partner” and “do it for him or her” • Helping the partner from the sidelines • Finding the equilibrium between the partner’s assistance and the couple relationship	• Facing together the fear of the progressive evolution of PD • Deep brain stimulation as an illusory hope

### A Closeness That Separates

Closeness makes it possible to compensate for the loss of autonomy, to anticipate the discomfort of the afflicted person and/or to support each other. For the dyads who lived independently, the disease imposes on this closeness and distorts the bond between the partners. Couples express this by using terms such as *a caregiver partner* (Bérengère and Bertrand) or an *infantilized partner* (Adrien and Aline), and some express rather definitive assessments regarding the relationship as a couple (“*I no longer have a husband*,” Bérengère; *“Bérengère, is a caregiver*,” Bertrand). Several relational dynamics explain how the link between partners weakens as PD progresses.

Camille and Claude illustrate a dynamic of *closeness/withdrawal-resignation:* Camille tries to get closer to her husband by providing assistance. If Claude consents to this help, it is difficult for him to accept how this emphasizes his dependence and he reacts by withdrawing emotionally, which was something that was already impinging on their relationship. This exacerbation of Claude’s withdrawal triggers a sense of resignation in Camille, and their relationship is gradually reduced to caregiving only.

**Table:** 

There is not really any sharing… He’s in his own world and it’s always been like that… the whole time we don’t really interact much in a meaningful way (Camille)	I depend more and more on my wife, and, out of necessity, I need her and I ask her for things that I did previously and that I did myself, so it brings us closer but at the same time it separates us (Claude)

A close, but more concrete, dynamic is seen with Domitille and David: *intrusion/withdrawal-impotence.* For couples accustomed to a degree of disparity in their communication of emotions, the dependence of the afflicted partner no longer allows them to evade the requests for sharing by the other partner. These requests become demands, experienced as an intrusion, since withdrawal from this loved one on whom they now depend is no longer possible.

**Table:** 

I am very mothering. My husband is someone who does not say much. It’s hard to have a discussion, to know what he thinks… (…) When things don’t go well, he doesn’t necessarily tell me, so as not to worry me… *I: But you see it. How does it work when it’s like that?* Well, I slowly drag it out of him (Domitille)	The hard thing is that she is always obliged to take care of me. Whether it’s to dress me, bath me, and such *I: And is that difficult for you, for her, or for both of you?* It’s hard for me to accept (Silence). Which makes me depressed (David)

Domitille’s mothering role allows David to express some of his suffering, but it no longer allows him to find solace in himself, and it traps him in a feeling of helplessness and despair regarding the situation.

The closeness of the assisting partner can also turn into aggressiveness, as expressed by Elisabeth and Eloi, in a dynamic of *closeness/attack blame.* Aggressiveness acts as an impediment to providing care and the need to share. This dynamic leads to a sense of helplessness and the distancing of the assisting partner as well as a feeling of guilt in the afflicted person, who burdens their partner with their unhappiness of seeing themselves as being dependent.

**Table:** 

My husband was a gentleman who was very calm. (…) He has become… there are times when what he says is outright mean. He never used to speak to me like that (Elisabeth)	We have more arguments than before (…) because I am always placing demands on her. In the end you become fed up. Also… it’s really difficult for her to have to cope with this… (Eloi)

On the other hand, for partners who were very close before the disease, as was the case for Fabienne and François, dependence consolidates a dynamic of *fusion/contagion-invasion*. The partners protect themselves by retreating into *being together in the “now.”* In this closeness, it is not possible to express one’s feelings without harming the other.

**Table:** 

I want to be with him for as long as possible (laughs), and then everything worries me, I mean… the… (silence) no I don’t want to talk about that (Fabienne)	At times she gets stressed and I think it’s a little bit because of me, when I say: *“me,”* it’s really me and the disease (François)

Thus, the disease is a theme that can be deliberately avoided for Gaëlle and Grégoire or Héloïse and Hugo. In these dyads, avoidance is observed at both the individual and the couple level, which can result in the alleviation of one’s own distress, the protection of the partner who is perceived as being vulnerable, and the preservation of the couple equilibrium.

### The Adversity Is Not Unbearable, but Going It Alone Would Be

Faced with pervasive symptoms soon after the diagnosis of the disease, Isabelle had been very attentive to supporting Isaac’s quality of life. The therapeutic education workshops allowed Isabelle to come to the realization that her interventions were excessive, that her partner relied too much on her and that they were not in keeping with her actual abilities. What Isabelle and Isaac now have in common is that they adjust their level of compensation for the symptoms as best as possible.

Julien explains how he manages to compensate for Juliette’s dependence, with the help of subterfuges, so that this diverted assistance does not reduce his wife’s experience or her efforts at autonomy. By being helped from the sidelines, she can try to preserve another equilibrium between the management of the disease and the preservation of an existential meaning. This same process could be seen with Kathy, who tries, very gently, to support and to assist, while helping Karim to preserve his place in the relationship.

**Table:** 

There is more of a complicity there are things that… I have to do, that I did not do previously (…) to try to accompany her, to help her… Quite often, it is when she is not there *I: Why?* I take her place when she is not there *I: Because if she is there it’s complicated?* It is she who is in charge (Laughs) (Julien)	I know we have to keep going, I’m still a joker. Whether with the children… you have to be able to laugh, to live… (Juliette)

Luc, in a relationship with Ludivine, is attentive but withdrawn and strives to find this same balance, stating resolutely that *“we should think about our lives, there are some really beautiful aspects, and that makes things go much better.”*

These couples are all aware of one last issue, the balance between assistance and their relationship with each other. Mélanie, whose partner is very disabled, explains the stress she faces between the *de facto* commitment to an asymmetrical support relationship and the choice of commitment to a relationship as a couple based on equality. Her husband, Marc, faced with the changes caused by the disease, highlights the importance of the emotional connection between the partners.

**Table:** 

In our relationship as a couple, we are equal, each with their differences, their strengths, their weaknesses, but we are also equal. While in a caregiver relationship, there is one who loses autonomy and the other compensates, and suddenly there is a question of… I wouldn’t necessarily say superiority, but… there is a power shift, and it is not always easy to reconcile that as a couple (Mélanie)	What I experienced quite soon was to say to myself, the hardship is not unbearable, it is not a disease, although it would be really tough to have to go it alone (Marc)

Thus, for couples who evolve as the disease intensifies, everyday life is the subject of a constant back and forth among these three issues: the control of the symptoms by the caregiver vs. by the person receiving the assistance, the management of the disease vs. the maintenance of an existential meaning, and providing assistance vs. a relationship as a couple. The success of these negotiations appears to be based on a degree of awareness of these issues by the partners, the symmetry in their respective efforts, and the extent of emotional support.

### Be Prepared for Anything and Facing an Uncertain Future

The interviews with Noémie and Nicolas show how the future mobilizes partners and the asymmetric regulation of the underlying feelings. Nicolas fears becoming a burden to his wife. Noémie was initially active by anticipating her husband’s loss of autonomy and by moving to suitable housing. She is now overwhelmed by the mental suffering of her husband, whose suicidal thoughts prevent her from expressing her own feelings.

**Table:** 

He said, one day if he realizes that he can no longer… he will put an end… *I: He will put an end to his days* So as not to make me suffer I know he would be capable of it. (…) That’s why I, the treatment, all that, I want to protect him (Noémie)	What will happen will happen, what do you expect me to say? (…) I will not be a burden to her! No! If I ever… *I: When you make this gesture like that across your neck, what does it mean?* (drily) it means what it means! If I become utterly useless? Hell no, I’m going to take care of things, as long as I am still able to! (Nicolas)

In contrast, for Bertrand and Bérengère, functioning is more symmetrical. They can imagine scenarios of a major loss of autonomy, and they share their outlooks, even regarding emotionally charged issues such as the end of life. They express the way they see the future, and they are aware of the decisions they will have to make.

**Table:** 

What scares me very much and would hurt me is to see him suffer. This we have already talked about. If he were just lying there, bedridden… If he can still think clearly and I see that he is begging me and that he is not well, I think I will go to Belgium *[note: a country that has legalized active euthanasia]* and I will have the courage (Bérengère)	There is not much of a way out for me, I have even contemplated suicide. If it ever gets worse, the situation I’m in right now, I’m going to ask for a favor… *I: Have you talked about this with her?* Yes, yesterday… Indeed yesterday We see it the same way… I wouldn’t be able to bear being taken care of the whole time… to be physically incapacitated (Bertrand)

Eloi and Elisabeth as well as Olivia and Oscar talk about the future by referring to the progress made possible by deep brain stimulation, a surgical procedure offered to patients who meet certain medical and psychological criteria when other treatments can no longer control the symptoms.

**Table:** 

Now with the neuro-stimulator, I hope it’s going to be slightly better for him, as well as that everything will work out… in order. (…) It will never be the same again. Because there is still the disease, it remains (Elisabeth)	It’s worth taking a look at all this… Now it is me who encourages her, I say: *“with this operation, it will be much better, we will regain the happiness we had before, find some enjoyment in life, have some cheer again in the house, watch TV until 11:00.”* (Eloi)

While the partners share the same hope, the symmetry is not total: Eloi and Oscar expect a spectacular improvement in their quality of life. Elizabeth has high hopes but also retains a degree of apprehension, while Olivia is more reserved and does not share her reservations.

In comparison, the experience of Isaac and Isabella illustrates a form of compromise. In a symmetrical manner, both express intense suffering in a scenario of a major loss of autonomy, but they are not oblivious to the future, and they are aware of what the medium term may bring. Isabelle envisions what may happen to her in the coming months, and she tries to cling to whatever good there is in the present. This ability to take into account the different aspects of the disease is a resource that makes it possible not to be consumed by a single very pessimistic outlook and not to be confined to avoidance.

**Table:** 

I am still worried. For me, it is an operation, which is not insignificant either… but I do not tell him that (Olivia)	I have talked to people about it… who were doctors and who are parkinsonians, they say it is a radical change. (…) I am amenable to anything to improve my daily life and that of my family (Oscar)

## Discussion

The objective of this study was to further understand couple mechanisms in a context where one of the partners has advanced-stage PD. In addition to the fact that the couple link is distorted as the disease progresses, this study identified different types of couple functioning that shed light on the mechanisms of the distancing or, on the contrary, the closeness of partners.

In the section “A Closeness That Separates,” couples expressed the experience of an asymmetrical relationship in which the afflicted partner loses their autonomy and feels dependent on the other, while the assisting partner assumes an important place in the relationship. As shown by Smith and Shaw (2017), this sense of interdependence awakens, on the one hand, a sense of closeness due to the illness and, on the other hand, a sense of frustration, which can be mutual, in light of the difficulty with preserving one’s autonomy. This ambivalence contributes to the emotional distance between partners. The roles of each partner are more pronounced, which can insidiously distort the bond of romance. Martin (2016) discusses the progression of the romantic relationship toward a “housemate” relationship. This study revealed this in the way each partner spoke of the other, relegating them either to a “caring partner” (the assisting partner) or an “infantilized partner” (the afflicted partner).

The types of dyadic functioning identified shed light on how couples try to find a new equilibrium. Each of the partners feels bound to the other but in a different way. While Martin (2016) showed the importance of caregiver support as a catalyst for closeness between partners, our study highlights novel aspects of the complex forms of this closeness.

In the first two typologies, the “closeness/withdrawal” and the “closeness/attack,” we note that the afflicted partner can distance themselves from this support provided by assuming a withdrawn or, by contrast, an aggressive position. This support can be a reminder for the afflicted partner of the painful observation of their loss of autonomy, as their body deteriorates and becomes uncontrollable (Maffoni et al., 2019). Couples engage in a constant struggle not to be overwhelmed by the sadness related to the progression of the disease. Distancing generates suffering in the assisting partner that they express through a feeling of resignation. The afflicted partner can be consumed with guilt, as they feel responsible for the suffering they sense their partner is going through. In the typology “fusion contagion-invasion,” the partners were already very close or even highly dependent on one another before the disease. The rigidity of this dyadic operation would allow them to set their fears aside to a certain extent. However, this symbiotic relationship stifles all individual expressions of emotions.

Through these couple dynamics, the insecurity caused by the progression of the disease exacerbates conflicts, as has already been highlighted in the literature (Maffoni et al., 2019), thus revealing all the couple distress experienced by these couples facing PD.

These patterns of functioning reveal the difficulty for the partners to free themselves from this vicious circle that gradually separates them at the emotional level. The second part, “The Adversity Is Not Unbearable, But Going It Alone Would Be,” illustrates how, for other couples, the awareness, at least partial, of this vicious circle allows them to wind back the relationship issues linked to the worsening of the symptoms. These couples, who otherwise might have allowed themselves to be overwhelmed by the disease, are more inclined to readjust and try to preserve their own identity, independent of their assigned role in the health process. This readjustment would make it possible, on the one hand, to alleviate the feeling of burden in the assisting partner (Tan et al., 2012) and, on the other hand, to maintain the autonomy and place of the afflicted partner outside their “status as a patient.” In these couples, the partners feel close to each other and display a sense of “collaboration” (Hodgson et al., 2004; Fergus and Skerrett, 2015; Smith and Shaw, 2017).

This awareness, even when partial, seems essential in the care process to help the couple readjust their couple dynamic to provide them with a greater degree of adaptability and flexibility and to leave room for emotional coregulation.

The future with the disease is one of the major challenges that partners face. In the third part, “Be Prepared for Anything and Facing an Uncertain Future,” the future was an issue that gave rise to couple concerns that, paradoxically, are in part managed individually.

For the afflicted partner, the fear of becoming a burden to the other is expressed in terms of suicide, medically assisted end of life, and liability. For some patients, thoughts of suicide are a way to try to regain control over the disease and to preserve a very weakened self-esteem. For others, anticipating the end of life allows them to come to terms with the prospect of finitude and to control the associated anxieties. When it has always been difficult for couples to share their feelings, the assisting partner, with the aim of protecting the other partner, may become overwhelmed with what they see as the difficulties ahead, well before the advanced stage, at the expense of physical and mental exhaustion. For other couples, the end of life is approached more openly, allowing each to express their fears and prepare advance directives.

Other afflicted partners envision a more optimistic scenario when they are offered the possibility of deep brain stimulation intervention due to the limitations of the previously tried treatments. For some patients, this procedure gives rise to a degree of hope that can blind them into thinking that they may be able to return to their former lives. The attitude of the assisting partner is generally more reserved or even withdrawn to avoid dashing the hopes of the afflicted partner of a better future while protecting themselves from the possibility of complications of the surgical intervention. These results are similar to those of Maffoni et al. (2019), who highlighted the commitment of the afflicted partner in a struggle between maintaining their past life and accepting the life imposed by the disease. It was noted that when it is possible to share fears, each partner can find a form of compromise by refocusing on the present, on what remains to be experienced, and not on what has been lost. This refocusing makes it possible to better accept the neurodegenerative and inescapable nature of the progression of PD, to readjust the present and to perceive the future in a different way by making PD part of it (Smith and Shaw, 2017; Maffoni et al., 2019).

The patient’s behaviors in the couple dynamic and the negative perception of the future for some patients could be associated with an anxious and/or depressive syndrome instead of PD progression. However, the clinical assessment of the most frequent PD-related neuropsychiatric disorders indicated a low level of depression, anxiety, and apathy in our sample. This result supports the idea that the patient’s position statement in the couple dynamic, whether more withdrawn or, in contrast, more aggressive, as well as the negative perception of the future was not associated with a psychiatric disorder but was more related to the lived experience of patients suffering from disease evolution.

Our study has several limitations that need to be considered for future research. First, our sample was mainly composed of male patients due to the higher prevalence of PD in men (e.g., Wooten et al., 2004). In future research, it would be useful to have a better balance in terms of the sex of the patients to explore whether sex makes a difference and how these differences are manifested at the individual and couple levels (e.g., Fleming et al., 2004).

Second, we chose to conduct separate interviews to allow each of the partners to freely express their feelings about their experience of the disease within their relationship as a couple. It would be interesting to study how couple dynamics are expressed concretely within a couple interview and how PD comes into play (Martin, 2016).

Third, this study sheds light on the importance of taking into account the history of the couple relationship to better understand the dysfunctional and resilient processes within the couple dynamic following the diagnosis of PD. Thus, it would be very useful to better understand the dynamic processes of the couple in its temporal dimension by meeting with the partners at different times of the disease.

This study provided a novel way of highlighting different patterns illustrating the dynamics of couples facing PD. Quantitative measures assessing the quality of the relationship (e.g., behavioral measures, questionnaires) could also be combined to quantify the representativeness of these different patterns with a larger sample. It would also be important to be able to measure the impact of these dynamics on the quality of life and the stress levels of partners using self-reported measures and how the clinical characteristics of the patients could explain some differences (e.g., Karlstedt et al., 2018).

Finally, from a theoretical point of view, the present research identified different concepts, such as individual and couple identity, empathy, couple attachment, and intimacy, through the exploration of patients’ and partners’ lived experience of the disease in their couple interactions. It would be interesting to extend the phenomenological research in this field to specific concepts associated with this population that is confronted with a vulnerable situation such as advanced PD, with the objective of considering the permanent interaction between the theory and clinical research in our understanding.

By understanding these dyadic processes even more precisely, specific clinical interventions could be provided at key stages of the disease to assist couples with coming to grips with changes in their individual and couple identities.

The results of this study confirm the significance of the marital subsystem (Wright, 2005) and integration of the assisting partner in the overall management of couples facing PD. Several clinical perspectives appear to be relevant. The history of the relationship as a couple appears to have a noticeable impact on the changes in the dynamics during disease progression. Clinical support inspired by narrative therapies (e.g., Kropf and Tandy, 1998; Caldwell, 2005) could also allow couples to generate a retrospective awareness of their conjugal relationship. The onset of the disease within their history as a couple could be an opportunity for partners to identify their individual and dyadic resources and to undertake a process of transforming emotional injuries prior to the diagnosis – injuries that may be unconsciously replayed in their couple dynamic.

Finally, emotionally focused therapy (EFT; Johnson, 2004) would be particularly appropriate to help couples become aware of the interrelational dynamics in which they find themselves. This approach would help them express their underlying feelings and needs so that the partners can understand each other better, readjust their behaviors, and thus reduce the feeling of individual and couple distress (Ghedin et al., 2017). Through this process of emotional reconnection, the assisting partner could assume more of this role as a caregiver by providing support to the afflicted partner without being perceived as overbearing. This safe space within the couple’s relationship would also allow the assisting partner to express their emotional feelings and relieve the feeling of guilt in the afflicted partner.

## Conclusion

This study confirms the importance of making the couple an integral consideration in the management of patients with PD. The study sheds new light on this process by focusing on the understanding of the positions taken by each of the partners in the couple dynamic and the processes of emotional regulation as PD progresses. Extending this study to different stages of the disease seems essential to further the understanding of the dyadic mechanisms underlying the adjustment of couples facing PD.

## Data Availability Statement

The original contributions presented in the study are included in the article, further inquiries can be directed to the corresponding author.

## Ethics Statement

The studies involving human participants were reviewed and approved by Committee for the Protection of Persons, CPP Est IV, IDRCB 2017-A0261152. The patients/participants provided their written informed consent to participate in this study.

## Author Contributions

EW, PA, KD, GB, and DL contributed to conception and design of the study. EC, EB, CS, CM, and BF realized the interviews and participated in the data analysis process. EC and EB wrote the first draft of the manuscript. All authors contributed to manuscript revision, read, and approved the submitted version.

## Conflict of Interest

The authors declare that the research was conducted in the absence of any commercial or financial relationships that could be construed as a potential conflict of interest.

## Publisher’s Note

All claims expressed in this article are solely those of the authors and do not necessarily represent those of their affiliated organizations, or those of the publisher, the editors and the reviewers. Any product that may be evaluated in this article, or claim that may be made by its manufacturer, is not guaranteed or endorsed by the publisher.
